# Temperature-Sensitive Hydrogels as Carriers for Modulated Delivery of Acetaminophen

**DOI:** 10.3390/gels9090684

**Published:** 2023-08-25

**Authors:** Snežana Ilić-Stojanović, Ljubiša Nikolić, Vesna Nikolić, Ivan Ristić, Suzana Cakić, Slobodan D. Petrović

**Affiliations:** 1 Faculty of Technology, University of Niš, Bulevar Oslobodjenja 124, 16000 Leskovac, Serbia; nljubisa@tf.ni.ac.rs (L.N.); nikolicvesna@tf.ni.ac.rs (V.N.); cakics@tf.ni.ac.rs (S.C.); 2 Faculty of Technology, University of Novi Sad, Bulevar Cara Lazara 1, 21000 Novi Sad, Serbia; ivan.ristic@uns.ac.rs; 3 Faculty of Technology and Metallurgy, University of Belgrade, 11000 Belgrade, Serbia; sloba@tmf.bg.ac.rs

**Keywords:** *N*-isopropyl acrylamide, 2-hydroxypropylmethacrylate, hydrogels, DSC, drug carrier, modulated drug release, acetaminophen

## Abstract

The purposes of this study are the polymerization of temperature-sensitive copolymers based on N-isopropyl acrylamide and 10 mol % of 2-hydroxypropylmethacrylate, characterisations of their thermal, morphological and swelling properties, as well as the analysis of potential application in drug-delivery systems. Acetaminophen, the representative of non-steroidal anti-inflammatory drugs, was used as a model drug in this study. It is a common pain relief drug, which is also used for fever treatment. However, oral administration comes with certain health risks, mainly the overdose and frequent administration of up to four times a day. The goal of applying temperature-sensitive hydrogel is to enable extended administration once a day, depending on the body temperature. The swelling behavior of the obtained poly(*N*-isopropyl acrylamide-*co*-2-hydroxypropylmethacrylate) (p(NIPA/HPMA)) hydrogels and their temperature-sensitivity, kinetics and order of swelling processes at 18 and 38 °C were analyzed. The thermal properties of these hydrogels were observed by the DSC method, and the obtained thermograms showed both melting and glass transitions. The drug delivery system of p(NIPA/HPMA) hydrogels with loaded acetaminophen was analyzed using scanning electron microscopy and Fourier transform infrared spectroscopy methods. Structural analysis of FTIR spectra indicates that non-covalent intermolecular interactions of the type of hydrogen bonds were formed among functional groups of acetaminophen and side-chains of p(NIPA/HPMA) hydrogels. The surface structure of p(NIPA/HPMA) hydrogels after drug loading indicates the acetaminophen presence into the pores of the hydrogel network, and their loading efficiency was higher than 92%. Qualitative and quantitative analysis of acetaminophen, determined by the high-pressure liquid chromatography method, showed that about 90–99% of the loaded amount was released from p(NIPA/HPMA) hydrogels within 24 h. Kinetic parameters of the acetaminophen release under simulated gastrointestinal conditions were determined. Based on obtained results, the drug delivery system of temperature-sensitive p(NIPA/HPMA) hydrogels with loaded acetaminophen could be suitable for additional investigation for modulated drug administration, e.g., for extended drug administration.

## 1. Introduction

Temperature-sensitive hydrogels belong to the wide group of stimuli-sensitive polymers, characterized by a cross-linked three-dimensional network obtained from synthetic or natural materials, which have the capability to swell while retaining plenty of liquid, without dissolving [1]. They are well-known as “smart” or “intelligent” hydrogels, because, with the temperature changes in the surroundings, they can respond with changing certain physicochemical characteristics [2]. Possible changes in its properties are, for example, swelling capacity, phase transitions, network structure, surface characteristics, permeability, mechanical properties, shape and volume, and they can turn back to their original state on the condition of elimination of the stimulus. Temperature-sensitive hydrogels are materials that are rationally designed, and they are broadly applied in human medicine and pharmacy [3,4,5]. The volume phase transition (VPT) in hydrogels was characterized by an immediate transformation in the swelling ratio [6,7]. Based on the volume phase transition and critical solution temperature, temperature-sensitive hydrogels can be divided into positively and negatively thermosensitive [8]. Negatively temperature-sensitive hydrogels possess a lower critical solution temperature (LCST), and they swell in the solution below critical temperature, while the hydrogel contracts above it [9]. The best-known negatively temperature-sensitive polymer is poly(N-isopropylacrylamide), which is widely researched and used to design many thermo-sensitive hydrogel systems [10]. It exhibits LCST in aqueous fluids at 32 °C, which is near the physiological body temperature [11,12,13]. Monomer *N*-isopropylacrylamide (NIPA) was usually copolymerized with numerous types of other synthetic monomers [14,15]), natural polymers or their combinations, in order to form the new, innovative hydrogels with different desired properties. Hydrogels have been utilized in many pharmaceutical and biomedical applications, for example, in temperature-sensitive modulated drug delivery [16,17,18,19], bioanalysis, bioseparations [20,21] and biosensors [22].

**Figure 1:**
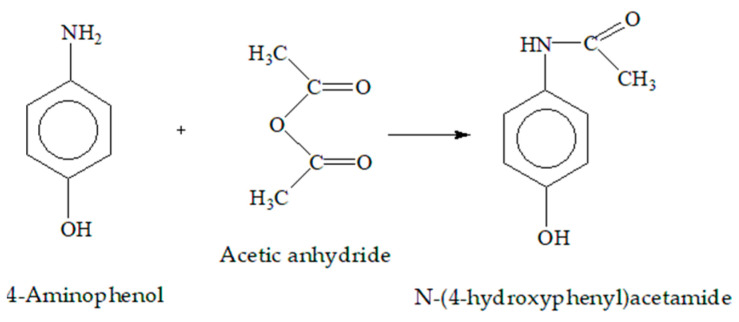
A possible route for acetaminophen synthesis from 4-aminophenol and acetic anhydride.

Acetaminophen, *N*-(4-hydroxyphenyl)ethanamide, is one of the most widely used pharmaceutical compounds, it belonging to the class of “aniline analgesics” [23,24]. It is a metabolite of phenacetin. A possible route for its synthesis from 4-aminophenol and acetic anhydride is presented on Figure 1 [25].

It shows different levels of analgesia, anti-inflammatory, antipyretic and antiplatelet activity [26,27,28]. Acetaminophen is a feeble inhibitor of two cyclooxygenase enzyme (COX-1, COX-2). It has no vasoconstrictive effect and is an effective in the treatment of acute, mild or moderate migraine attacks [29]. Its half-life is 1–4 h, and is increased in cases of liver damage and overdose patients [30]. It has been recently used in the treatment of neurodegenerative diseases, such as Alzheimer’s disease, protecting the brain’s endothelial cells from oxidative stress [31]. N-acetylcysteine can be used as an antidote in cases of acetaminophen overdose [32]. According to the Biopharmaceutical Classification System (BSK/BSC), i.e., the guide for predicting the intestinal absorption of drugs, acetaminophen belongs to class I (highly soluble and highly permeable) medicinal substances [33]. With oral administration, there are certain health risks of overdose and frequent administration of up to four times a day. Orodispersible hydrophilic wax tablets have been formulated to increase the solubility and bioavailability of acetaminophen [34]. Micronization of acetaminophen particles with supercritical carbon(IV)-oxide was also performed, resulting in particles with a finer structure and greater bioavailability [35]. Many researchers investigated the possibility of using intelligent gels as carriers of acetaminophen for its modified/controlled release. Multifunctional copolymer hydrogels of *N*-vinyl-2-pyrrolidone and poly(acrylic acid) were tested as acetaminophen and aspirin carriers, but without analysis of their release mechanisms [36]. Kinetics of swelling and release of acetaminophen from pH- and temperature-sensitive hydrogels based on sodium alginate and NIPA under simulated gastric conditions indicate anomalies in the transport mechanism and a good fit with a first-order kinetic model [37]. The effect of the composition of hydrogels on the swelling process and release of acetaminophen in aqueous media of different pH values from poly(acrylamide-*co*-itaconic acid) was also investigated [38]. Tests have shown that the functional monomer 4-[(4-methacryloyloxy)phenylazo]benzenesulfonic acid with poly(acrylamide) hydrogel provides the affinity of the hydrogel to acetaminophen, whose release can be photoregulated, but without the release mechanism analysis [39]. The acetaminophen release from poly(2-hydroxyethyl methacrylate-*co-N*-vinyl-2-pyrrolidone) hydrogels at 37 °C showed a different rate and release mechanisms due to weaker interactions of the polymer with water at pH = 7.2 in the simulated intestinal fluid, SIF, compared with the simulated gastric fluid, SGF, at pH = 1.2 [40]. The mathematical modelling of releasing the acetaminophen from gel based on hydroxypropylcellulose and poly(acrylamide), 25/75 wt %, was analyzed using a genetic programming algorithm [41]. The obtained results showed that a greater amount of acetaminophen was released at pH = 7.38 than at pH = 7. Polyurethane nanocomposite hydrogels with organofillized montmorillonite were applied as matrix for analysis of the acetaminophen release process [42]. The temperature increases at 37 °C speed up the acetaminophen release process compared with the results at 23 °C. Poly(*N*-isopropylacrylamide-acrylamide) membrane was applied in order to study of the mechanism of both vitamin B12 and acetaminophen transport through hydrogels at phosphate buffer solution (pH 7.4) and different temperatures [43]. Biodegradable composites obtained from polyvinyl alcohol in combination with nanofibrillated cellulose and 2,2,6,6-tetramethylpiperidine-*N*-oxyl-oxidized nanofibrillated cellulose showed a release of acetaminophen only about 14% from nanofibrillated cellulose, and about 28% from 2,2,6,6-tetramethylpiperidine-*N*-oxyl-oxidized nanofibrillated cellulose with the occurrence of a burst release from the outer film layer, during 6 days [44].

Based on presented results in available literature, there is no single temperature sensitive carrier which could be suitable for extended release and administration once a day. For this reason, the goal of this study is the characterization of hydrogels poly(*N*-isopropyl acrylamide-*co*-2-hydroxypropylmethacrylate), p(NIPA/HPMA), as potential carriers of acetaminophen. The profiles of drug delivery from corresponding hydrogel network systems were monitored in previous studies for the investigation of possible controlled release of some NSAID, e.g., caffeine [45], piroxicam [46], naproxen [47], phenacetin (as a structural analog of acetaminophen) [48] and ibuprofen [49]. Based on the previous studies, the major hypothesis of the current study was that similar “intelligent” p(NIPA/HPMA) hydrogels will improve the acetaminophen release mechanism with benefits over previous results. Not one comparable investigation mentioning hydrogel networks as carriers for modified acetaminophen release was found in the available publications.

## 2. Results and Discussion

### 2.1. Synthesis of p(NIPA/HPMA) Hydrogel

The process of obtaining p(NIPA/HPMA) hydrogels consisted of 10 mol % of comonomer 2-hydroxypropylmethacrylate (HPMA) calculated in relation to the initial NIPA monomer amount and 0.5, 1.0, 1.5, 2.0 and 3.0 mol % of ethyleneglycoldimethacylate (EGDM) as crosslinker. The probe with 0.5 mol % of EGDM crosslinker maintained the liquid structure after synthesis without the need for hydrogel consistency, and, for that reason, it was excluded from further analysis. The other samples of obtained hydrogels were extracted unreacted molecules during the polymerization process and, after that, they were dried in the xerogel state for further analyses, analogous to the procedure described in the author’s previous studies [9,46,47,50].

### 2.2. Differential Scanning Calorimetry

**Figure 2:**
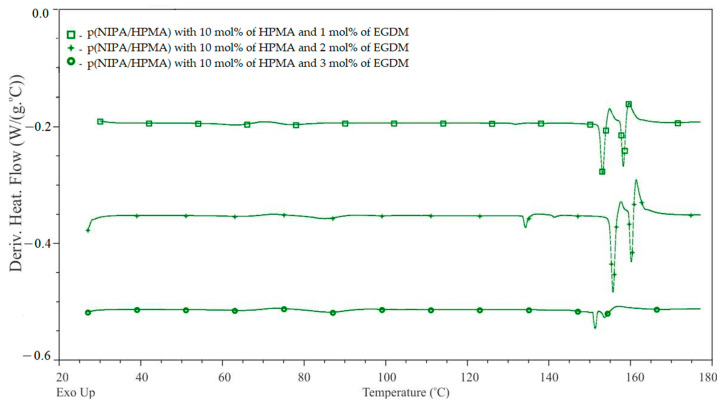
The first derivative of heat flow of the p(NIPA/HPMA) xerogels with 10 mol % of HPMA and 1.0, 2.0 and 3.0 mol % of ethyleneglycoldimethacylate.

The thermal phase transitions of obtained p(NIPA/HPMA) hydrogels with 10 mol % of HPMA and with 1.0, 2.0 or 3.0 mol % of the ethyleneglycoldimethacylate were detected in differential scanning calorimetry (DSC) thermogram. The first derivative of heat flow in the range of 30–180 °C is shown in Figure 2. It shows two sharp peaks from melting of ordered structures and also the glass transitions with weak intensity.

Values obtained from DSC thermogram, including the melting temperatures (*T*_m_), the melting enthalpy and the glass transition temperatures (*T*_g_) for the obtained p(NIPA/HPMA) xerogels are presented in Table 1.

Data obtained from DSC thermogram for the p(NIPA/HPMA) xerogels with 1, 2 and 3 mol % of ethyleneglycoldimethacylate shows that the melting process happens in two intervals (first from temperature 153.90 °C to 156.49 °C and second from 156.98 °C to 160.73 °C). The melting enthalpy is in the range of 2.79–9.85 J∙g^−1^. When the hydrogel is heating, its crystalline regions become disordered and pass through a viscous liquid phase, which causes transformation of its physical state, characterized as the melting temperature. The hydrogel network structure has different distances between crosslinking nodes, divergent branching and chain length, with various melting temperatures. This is the reason for its broader melting temperature and varied enthalpy. These results also confirmed the suitable miscibility and compatibility of the monomers NIPA and HPMA, as well as structural irregularity [9,44,51]. Synthesized hydrogels are stable up to temperatures of about 150 °C. The common sterilizing temperatures are in the range of 121–132 °C. Obtained hydrogels are convenient for sterilizing and enable biomedical application as drug carriers.

The DSC analysis of p(NIPA/HPMA) xerogels also provides results of the glass transition temperatures. The glass transition is the characteristic process for amorphous regions of the hydrogels during its heating, when it changes from a glassy to a soft state. This state is usually named as “viscous liquid”. However, since it has not been melted yet, the temperature range of its occurrence depends on the hydrogel chemical structure. The samples with 2.0 and 3.0 mol % of ethyleneglycoldimethacylate display three glass transition temperatures, while the sample with 3 mol % of ethyleneglycoldimethacylate displays two glass transitions temperatures. The first occurrence of the glass transition temperatures is in the range 63.41–64.79 °C, the second is in the range 77.53–86.47 °C and the third is in the range 134.81–131.76 °C. Some difference in the glass transition temperatures between hydrogels with different molar mass of the crosslinker are visible. These differences are probably consequences of diverse crosslinking density, conditioned from the content of ethyleneglycoldimethacylate, and show similarity to previously published works [46,51].

DSC analysis confirmed the amorphous, semi-crystalline structure of the synthesized p(NIPA/HPMA) hydrogels.

### 2.3. Swelling Behavior

#### 2.3.1. Temperature Sensitivity Analysis

**Figure 3:**
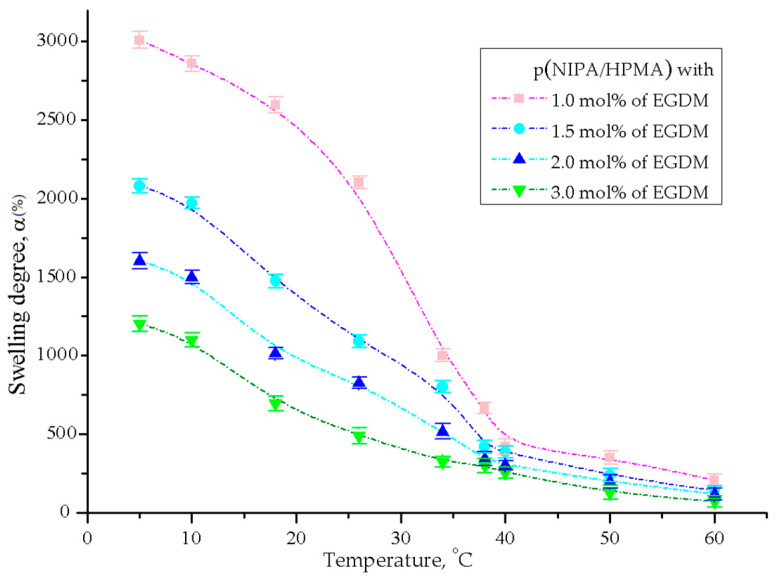
Temperature sensitivity of the p(NIPA/HPMA) xerogels with 10 mol % of HPMA and 1.0, 2.0 and 3.0 mol % of ethyleneglycoldimethacylate. Error bars represent the standard deviation of three replicates.

The swelling behavior of p(NIPA/HPMA) xerogels with 10 mol % of HPMA in distilled water was monitored during heating from 5 to 60 °C to examine their temperature sensitivity. Results of the equilibrium swelling degree, α_e_, calculated using Equation (1), for p(NIPA/HPMA) xerogels as a function of the temperature are presented in Figure 3.

All p(NIPA/HPMA) hydrogel samples show sensitivity to temperature changes. The greatest swelling degree at all tested temperatures was achieved at lower temperatures (from 5 to about 20 °C) by the sample with 1 mol % of ethyleneglycoldimethacylate [9,46,50]. Significant reduction of the swelling degree happens when temperature increase in the range of 30–38 °C, when hydrogels pass a volume phase transition temperature (VPTT) which is named as the lower critical solution temperature (LCST). When the temperature exceeds 50 °C, these dependencies asymptomatically approach a constant value. As expected, the swelling degree increased with decreasing crosslinking ratio, because the smaller number of crosslinker decreases the network density, increasing the elasticity and mobility of the polymer chains and pore size. At temperatures below LSCT, hydrogels are swollen, soft and transparent. However, at temperatures above the LCST, they deswell, reducing their volume and changing to opaque white and rigid due to the breaking of intermolecular hydrogen bonds [46,51]. The hydrogen bond formation between the amide and hydroxyl groups of hydrogel and water molecules causes both hydration and swelling, while the amide–amide hydrogen bond formation led causse dehydration; therefore, they have a potential role in the phase transition. When the temperature rises above the LCST, intermolecular hydrogen bonds break up, and hydrophobic interactions cause the prevention of the enlargement of hydrogel network and water molecules ejectment becomes dominant [48,52]. The swelling behavior of p(NIPA/HPMA) hydrogels exhibited a resemblance to the results of the comparable hydrogels available in the literature [9,46,47,49,50,53]. The exhibited swelling behavior of synthesized hydrogels is known as negatively thermo-sensitive, which is very useful for efficient drug absorption at a temperature below the LCST, and drug delivery when temperature rises above the LCST.

#### 2.3.2. Analysis of the p(NIPA/HPMA) Hydrogel Swelling Kinetics

**Figure 4:**
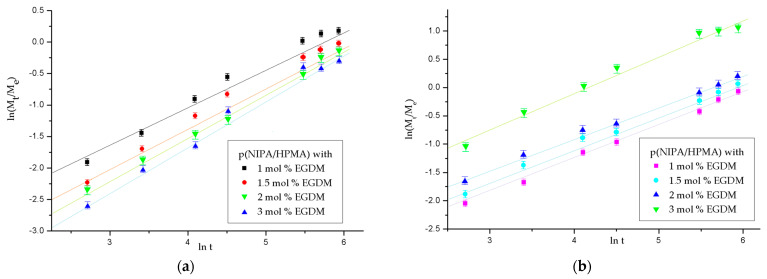
Kinetic parameters n and k for series of p(NIPA-HPMA) hydrogels with 10 mol % HPMA at: (**a**) 18 °C and (**b**) 38 °C. In each figure, error bars represent the standard deviation of three replicates.

The equilibrium swelling degree, *α_e_*, values for p(NIPA/HPMA) hydrogels at 18 °C and 38 °C in the fluid with pH value 7.4, and kinetic parameters (the diffusion coefficient, diffusion exponent, the correlation coefficient (R^2^) and kinetic constant,) calculated with expressions (2)–(6), are presented in Figure 4 and in Table 2.

Based on the swelling kinetics results at pH = 7.4 (Figure 4a,b and Table 2) and especially the diffusion exponent, *n*, values, the transport mechanism into the p(NIPA/HPMA) hydrogels samples at both temperatures (18 °C and 38 °C) corresponds to “non-Fickian diffusion” for all samples and contributes to the water-sorption process. The fluid transport into the p(NIPA/HPMA) network exhibited an anomaly in the fluid transport mechanism, i.e., the swelling mechanism was controlled by diffusion of fluid and relaxation of macromolecular chains and did not obey the rules of the Fickian law. The fluid transport is complicated by segmental mobility retardation, molecular relaxation, crystallization, functional interaction among penetrant and macromolecules, etc. Non-Fickian diffusion was characterized by an initial induction time, when the sharp front is established near the film surface, which separates the highly swollen area from a dry, glassy area, and the amount of absorbed fluid increases linearly with time [54]. Comparable results for the diffusion exponent of hydrogels p(NIPA/HPMA) at 5, 25, and 38 °C have been previously published [32,46,47]. Increasing the crosslinker content and enhancing the network density leads to a reduction in the value of n, which means that the swelling degree decreases [47,48].

The kinetic constant, k, values are slightly smaller at 18 °C relative to data at 38 °C due to decrease of the free volume above the LCST, after the process of shrinking of the macromolecular chains [47,50].

The diffusion coefficient, *D*, values (calculated for the initial phase of swelling to 60%) at 18 °C are in the range 0.397 × 10^−6^–8.998 × 10^−6^ cm^2^/min. Above the LCST, at 38 °C, the diffusion coefficient values are in the range of 0.676 × 10^−5^–3.102 × 10^−5^ cm^2^/min, and indicate faster diffusion and growth in the values for all analyzed samples of p(NIPA/HPMA) hydrogels. The increased values of the *D* (diffusion coefficient) with the increase of temperature, showed known effect of temperature on sigmoidal non-Fickian diffusion process, also described in the literature [46,47].

#### 2.3.3. The Order of the Swelling Reaction

**Figure 5:**
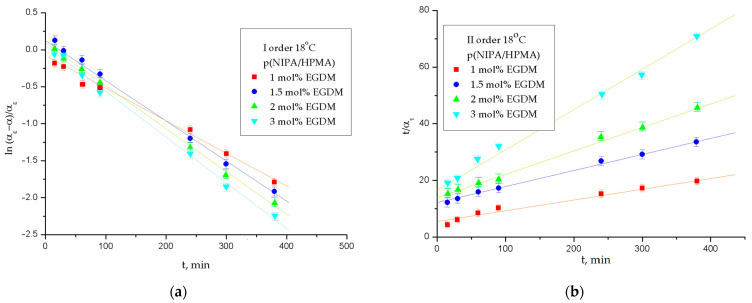
Determination of the reaction order of the swelling process for series of p(NIPA/HPMA) hydrogels during 6 h at 18 °C (**a**) first order; (**b**) second order. In each figure, error bars represent the standard deviation of three replicates.

**Figure 6:**
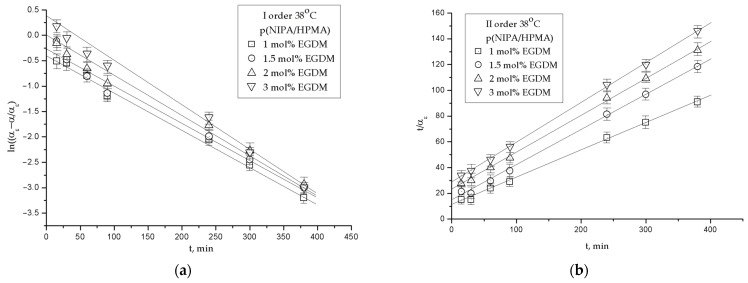
Determination of the reaction order of the swelling process for series of p(NIPA/HPMA) hydrogels during 6 h at 38 °C: (**a**) first order; (**b**) second order. In each figure, error bars represent the standard deviation of three replicates.

The experimentally obtained and calculated normalized equilibrium swelling ratio values, the rate constant for I-order and II-order and the linear correlation coefficient values, R^2^, for p(NIPA/HPMA) hydrogels were obtained by applying Equation (7) for the first order and Equation (8) for the second order of the swelling reaction at 18 and 38 °C (Table 3 and Figure 5 and Figure 6).

The calculated and experimentally obtained values of normalized equilibrium swelling degree for p(NIPA/HPMA) hydrogels at the temperature of 18 °C were in a concordance (Table 3 and Figure 5). The values of the linear correlation coefficient, R^2^, are nearly 1, and designate good agreement of the experimental values with the supposed order of swelling reaction. However, they are more suitable for the second-order reaction.

The calculated and experimentally obtained values of normalized equilibrium swelling degree for series of p(NIPA/HPMA) hydrogels at 38 °C (Table 3, Figure 6) showed similarity with data at the lower temperature (18 °C).

The calculated values of normalized equilibrium swelling degree and experimentally obtained at 38 °C for p(NIPA/HPMA) hydrogels were in agreement. The values of the linear correlation coefficient, R^2^, are also close to 1, and indicate good a of the experimental results with the assumed swelling reaction order, but they correspond better to a second-order reaction. The values of the reaction rate constants, *K*, for all samples are within the same order of magnitude (Table 3). It might be said that the swelling process of p(NIPA/HPMA) hydrogels demonstrated a little higher deviation from the first order of the swelling mechanism at both tested temperatures (18 °C, 38 °C). Consequently, it could be concluded that the swelling process of p(NIPA/HPMA) hydrogels obeys the second order of the swelling reaction, which is comparable to the one described in previous work for analogous hydrogels [46].

### 2.4. Fourier Transform Infrared Spectra

#### 2.4.1. FTIR Spectrum Analysis of Acetaminophen

**Figure 7:**
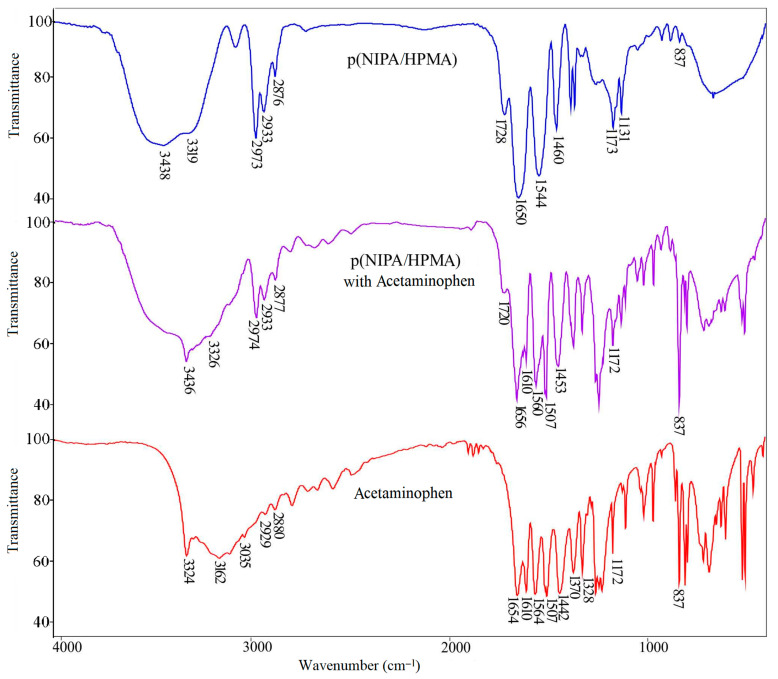
FTIR spectra of: synthesized p(NIPA/HPMA) hydrogel, p(NIPA/HPMA) hydrogel with loaded acetaminophen and acetaminophen.

The FTIR spectrum of acetaminophen is shown in Figure 7, and the wavenumber maximums values of the characteristic absorption bands are given in Table 4. In the FTIR spectrum of acetaminophen, there is an absorption band of medium strength, originating from the stretching vibrations of the amino group of the secondary amide, ν(NH), with an absorption maximum at 3324 cm^−1^. The broad band with a maximum at 3162 cm^−1^ originates from the stretching vibrations of the phenolic hydroxyl group, ν(Ar-OH). The stretching C-H vibrations of the phenyl group, ν(C-H), give bands of weak intensity with a maximum at 3035 cm^−1^. The stretching C-H vibrations of the methyl group give bands of weak intensity originating from ν_as_(CH_3_) with a maximum at 2929 cm^−1^ and ν_s_(CH_3_) with a maximum at 2880 cm^−1^, as well as bending vibrations of medium intensity, δ_s_(CH_3_), whose bands appear in the spectrum of acetaminophen at 1370 cm^−1^ and 1328 cm^−1^. The stretching vibrations of the C=O group from the secondary amide, ν(C=O), are characterized by a strong band appearing in the spectrum with a maximum at 1654 cm^−1^. The maximum of the “amide band II” from the in-plane bending vibrations of the -NH group, δ(N-H), occurs at 1564 cm^−1^ in the spectrum of acetaminophen. The out-of-plane bending vibrations, γ(C-H), characteristic of para-disubstituted benzene, give a sharp band with a maximum at 837 cm^−1^. The skeletal vibrations of the C=C bond from the phenyl group in the spectrum give characteristic strong bands with maximum at 1610 cm^−1^ and 1507 cm^−1^ [55].

#### 2.4.2. FTIR Spectrum Analysis of p(NIPA/HPMA) Hydrogel with Loaded Acetaminophen

In the FTIR spectrum of the p(NIPA/HPMA) hydrogel with loaded acetaminophen (Figure 7 and Table 4), the absorption maximum from the stretching vibrations of the -OH group, ν(OH), can be observed at 3436 cm^−1^, which was moved by 2 units toward lesser wavenumbers relative to the location in the hydrogel. In the FTIR spectrum of the p(NIPA/HPMA) hydrogel with loaded acetaminophen, the band of stretching vibrations of the N-H group, ν(NH), appears at 3326 cm^−1^ and was moved to higher wavenumbers by 7 units compared with the p(NIPA/HPMA) hydrogel, and by 2 units compared with acetaminophen. These shifts and the presence of a broadened band in the part around 3400 cm^−1^ indicate the formation of intermolecular hydrogen bonds among the chains of the p(NIPA/HPMA) hydrogel and acetaminophen NH•••O and OH•••O, which is also confirmed in the previous investigation [56]. In the FTIR spectrum of the p(NIPA/HPMA) hydrogel with loaded acetaminophen, there are no bands from the stretching vibrations of the C-H group, ν(C-H), and -OH from the phenolic group of acetaminophen, ν(Ar-OH), and it is considered that they are covered by the C-H vibrations from the p(NIPA/HPMA). “Amide band I”, ν(C=O), appears in the spectrum of the p(NIPA/HPMA) hydrogel with loaded acetaminophen at 1656 cm^−1^ and was moved towards higher wavenumbers by 6 units in relation to the position in the spectrum of the hydrogel, and by 2 units in relation to position in the spectrum of acetaminophen. The maximum shift of the “amide band II”, δ(N-H), which occurs at 1560 cm^−1^ in the spectrum of the p(NIPA/HPMA) hydrogel with loaded acetaminophen, by 16 units to higher wavenumbers compared to the position in the spectrum of the hydrogel and by 4 units towards lower wavenumbers compared to acetaminophen, confirms that the -NH group participates in the hydrogen bond construction. The band with the maximum at 1720 cm^−1^ in the spectrum of the p(NIPA/HPMA) hydrogel with loaded acetaminophen originates from the stretching vibrations of the keto group from the ester part of the structure, ν(C=O), and was shifted to lower wavenumbers by 8 units compared with the hydrogel, which indicates that it participated in the formation of intermolecular hydrogen bonds. The maximum of the band of hydroxyl group bending vibrations, δ(OH), in the spectrum of the p(NIPA/HPMA) hydrogel with loaded acetaminophen, occurs at 1453 cm^−1^ and was shifted by 11 units towards higher wavenumbers in relation to the position in the spectrum of acetaminophen, which confirms that the -OH group participates in building a hydrogen bond. The presence of acetaminophen within the p(NIPA/HPMA) hydrogel structure is confirmed by the appearance of characteristic bands’ maximum values from the stretching vibrations of the C=C bond from the aromatic structure at 1610 and 1507 cm^−1^. Small shiftings between p(NIPA/HPMA) copolymer and acetaminophen suggest that the mentioned interactions are of the non-covalent type. The specified shifts of the individual bands maximum values indicate the formed intermolecular interactions of hydrogen bond type between the hydrogel and acetaminophen over hydroxyl-, ester- and amino- groups. These studies are in agreement with available literature data [36,37].

The corresponding FTIR spectrum of p(NIPA/HPMA) was analyzed in the previously mentioned paper by the authors [48] and used for this investigation.

The structural variations in the p(NIPA/HPMA) hydrogel with loaded acetaminophen are given as the FTIR spectrum in Figure 7, and the wavenumber maximum shifts of the characteristic absorption bands are given in Table 4.

### 2.5. Acetaminophen Loading Efficiency into p(NIPA/HPMA) Hydrogels

The loaded amount of acetaminophen into the three-dimensional networks of p(NIPA/HPMA) hydrogels was calculated based on the difference in sample weights before and after the loading process, i.e., its swelling in the acetaminophen solution. The acetaminophen loading efficiency into the p(NIPA/HPMA) hydrogels, obtained by applying Equation (9), is shown in Table 5.

The available amount of acetaminophen for loading into each sample of p(NIPA/HPMA) hydrogels was in the range of 92.16–96.06% and confirms excellent loading efficiency.

**Figure 8:**
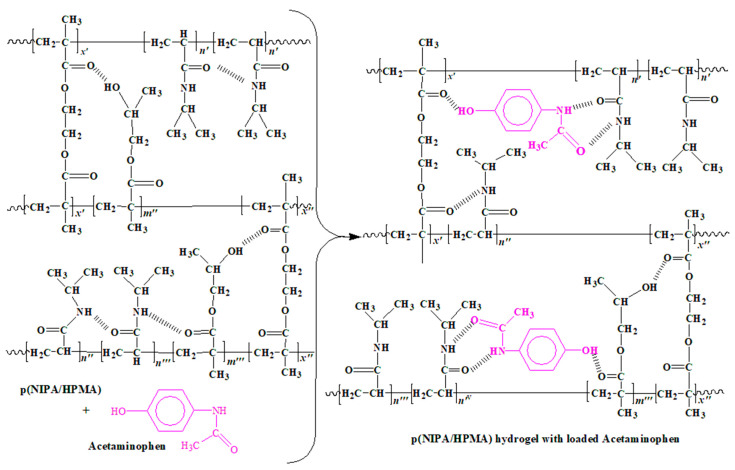
Drug delivery system based on acetaminophen (in the pink color) loaded into the p(NIPA/HPMA) hydrogels (the possible intramolecular interactions are designated).

The potential process of loading acetaminophen into the network structure of p(NIPA/HPMA) hydrogels with the possible formation of non-covalent hydrogen bonds between drug and lateral functional groups from hydrogel, according to FTIR analysis, is schematically presented in Figure 8.

### 2.6. Morphology Characterization

**Figure 9:**
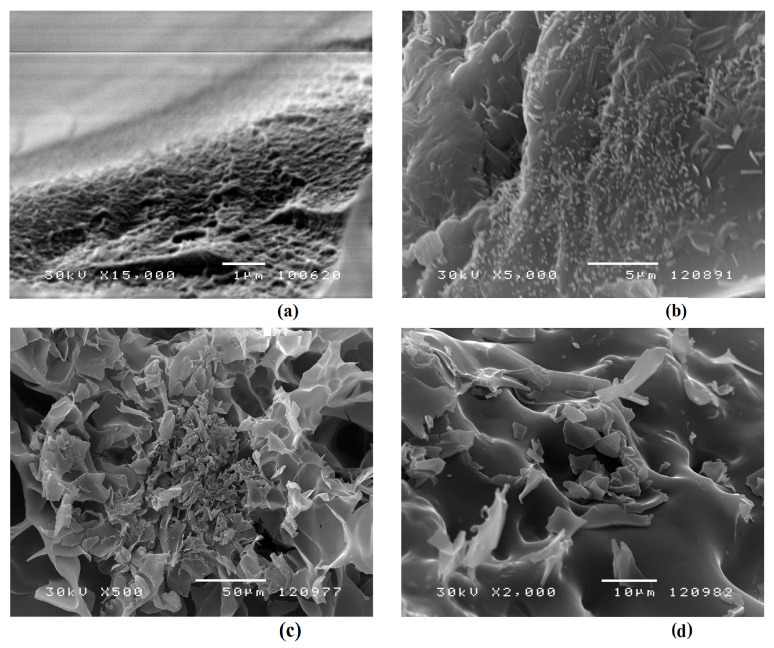
SEM micrographs of: (**a**) the p(NIPA/HPMA) in xerogel state, magnification 15,000× *g*, scale bar 1 μm; (**b**) p(NIPA/HPMA) with loaded acetaminophen in the xerogel state, magnification 5000× *g*, scale bar 5 μm; (**c**) p(NIPA/HPMA) with loaded acetaminophen in the equilibrium swollen state, magnification 500× *g*, scale bar 50 μm, (**d**) p(NIPA/HPMA) with loaded acetaminophen in the equilibrium swollen state, magnification 2000× *g*, scale bar 10 μm.

SEM micrographs of p(NIPA/HPMA) xerogels without and with loaded acetaminophen are shown in Figure 9. The microstructure of the p(NIPA/HPMA) xerogel after synthesis confirms the effect of copolymerization on gel morphology. It shows numerous micro-cracks and micro-pores. The pore diameters of the p(NIPA/HPMA) xerogel ranged from 0.2–0.80 μm, with an average pore diameter of 0.5 μm (Figure 9a) with interconnecting cracks. The pore distribution on the micrograph (Figure 9a) showed the presence of large pore diameters, which contribute to the drug diffusion process as much as the smaller area of the smaller pores.

The surface structure of p(NIPA/HPMA) with loaded acetaminophen in the xerogel state (Figure 9b) indicates the incorporation of acetaminophen inside the pores because it is significantly different compared with the structure of the empty xerogel (Figure 9a). Crystals of acetaminophen that are observed on the surface show similarity to the ones discussed in the previously published works [35].

A clearer representation of the microsurface of p(NIPA/HPMA) hydrogel with loaded acetaminophen (swollen to equilibrium and freeze-dryed) is shown in Figure 9c,d. In these micrographs, the change in the hydrogel network structure is clearly visible, which is caused by the semi-homogeneous arrangement of acetaminophen crystals within the three-dimensional p(NIPA/HPMA) hydrogel network. The surface of p(NIPA/HPMA) with loaded acetaminophen indicates a nonuniformal dispersal of acetaminophen inside the hydrogel network. Certain areas are abundant with acetaminophen crystals that are retained on the three-dimensional microsurface. During the delivery process, they are the first to leave the network structure.

Based on the results obtained by the FTIR and SEM methods, as well as the acetaminophen loading efficiency, it can be concluded that acetaminophen has shown excellent loading in the p(NIPA/HPMA) hydrogels, especially in the sample with 1 mol % of crosslinker, thanks to the weak interactions between the electronegative oxygen from -OH and the ester group C=O or nitrogen from the -NH group.

### 2.7. In Vitro Acetaminophen Delivery from p(NIPA/HPMA) Copolymers

**Figure 10:**
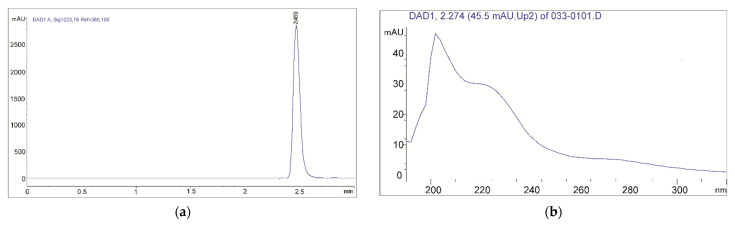
Chromatogram of acetaminophen from RP HPLC (**a**) with UV spectrum from DAD detector (**b**).

The amount of released acetaminophen from p(NIPA/HPMA) hydrogels was determined by Equation (10), which corresponds to the linear part of the constructed calibration curve. The maximum in the HPLC chromatogram at a retention time Rt = 2.469 min (according determined RP HPLC settings) originates from acetaminophen, and it is presented in Figure 10a.

The UV spectrum of acetaminophen, obtained by recording on a DAD detector, has shown two characteristic absorption maxima (Figure 10b). The primary maximum at λ_max_ = 205 nm originates from the π→π* transition of C=C bonds from benzene, and the secondary one with a band at λ_max_ = 230 nm is the result of the π→π* transition from the keto group. The bathochromic shift of the primary and secondary bands is the result of the conjugation of the unbound electrons of the substituent -OH and -NH groups with the π electrons of benzene [57].

**Figure 11:**
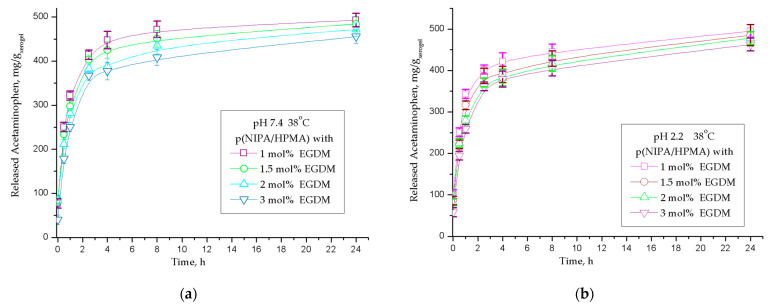
In vitro cumulative acetaminophen release from series of p(NIPA/HPMA) hydrogels during 24 h at 38 °C at: (**a**) pH 7.4, (**b**) pH 2.2. In each figure, error bars represent the standard deviation of three replicates.

In vitro cumulative acetaminophen release from the synthesized p(NIPA/HPMA) hydrogels at 38 °C during 24 h in the acidic fluid (with pH values of 2.2) and in the alkaline fluid (pH = 7.4) are given in Figure 11.

The content of released acetaminophen in the simulated gastrointestinal conditions (pH = 2.2 and pH = 7.4 at 38 °C) from samples of copolymer p(NIPA/HPMA) hydrogels is shown in Figure 11a,b, respectively. It can be noted that the p(NIPA/HPMA) hydrogel with the lowest crosslinking (sample with 1 mol % of crosslinker ethyleneglycoldimethacylate) released the highest quantity of acetaminophen: 492.77 mg/g_xerogel_, or 98.56% of the loaded quantity at pH = 7.4, and 480.16 mg/g_xerogel_, i.e., 96.03% at pH = 2.2 (Table 6). The smallest acetaminophen quantity was released from the sample with the highest crosslinking density (with 3 mol % of ethyleneglycoldimethacylate): 455.59 mg/g_xerogel_, or 91.12% of the loaded quantity at pH = 7.4 and 451.58 mg/g_xerogel_, i.e., 90.31% at pH = 2.2. The analysis of the obtained results shows a high similarity in the amount of acetaminophen released from all hydrogel samples under conditions with different pH values. Small differences in the quantity of released acetaminophen in an acidic environment compared with the weak alkaline fluid can be attributed to the influence of ionization of the -OH group of acetaminophen, because in the molecular structure of p(NIPA/HPMA) hydrogels, there are no functional groups that could be ionized in aqueous solution. The probable reason is an increase in network density and a smaller internal free volume that can accommodate the drug, which is in agreement with the swelling results. At 38 °C, which is above the LCST (and also the internal physiological body temperature), the p(NIPA/HPMA) hydrogels are in a contracted state because they undergo the phase transition. The intermolecular hydrogen bonds between the drug and the hydrogel are broken at this temperature, and acetaminophen is released from the hydrogel network. The drug release rate from the macromolecular network is dependent on the intermolecular bonds among the acetaminophen and the lateral groups of the hydrogel, which was also shown in the studies of other authors [36,38].

The mechanism of acetaminophen diffusion from p(NIPA/HPMA) hydrogels was assessed by fitting results of experimental release using Equations (4)–(7), and the kinetic parameters (*n*, *k* and *D*) are all presented in Table 6.

The kinetic parameters of the acetaminophen release from the p(NIPA/HPMA) hydrogels at slightly alkaline conditions (at pH 7.4) and only the sample with 1 mol % of crosslinker in acidic fluid (at pH 2.2) display that the mechanism of fluid transport belongs to Fickian diffusion, characterized by a fluid permeation process which is considerably slower compared with the relaxation of the macromolecular chains and is controlled by the diffusion (Table 6). Obtained results of kinetic parameters of the acetaminophen released from these hydrogels at acidic fluid (at pH 2.2) for samples with 1.5, 2 and 3 mol % of crosslinker show that the fluid transport mechanism follows non-Fickian diffusion process. Values of the diffusion coefficient, D, from all analyzed p(NIPA/HPMA) hydrogels were similar and show slight variations between samples.

It is notable that approximately full quantity of loaded acetaminophen (about 90–99%) was delivered during the first 24 h and only 1–10% persisted within the hydrogel’s network. This result provides the possibility that the analysed drug delivery system based on acetaminophen and synthesized p(NIPA/HPMA) hydrogels could be suitable for extended release and administration once a day. The average adult dosage for fever and pain may be given depending on the formulation: parenterally every 4 h, orally immediate-release every 4 to 6 h or extended-release orally every 8 h [58,59]. Design of the new drug delivery system could be easier for administration, in comparison with conventional therapy. The advantages of the current study are evident after comparison with the published acetaminophen polymeric carriers mentioned in the introduction, which are presented in Table 7.

The obtained results presented in this study show that thermosensitive p(NIPA/HPMA) hydrogels with acetaminophen would be of interest as drug delivery systems for further testing, especially for extended acetaminophen release.

## 3. Conclusions

Temperature-sensitive p(NIPA/HPMA) hydrogels with 10 mol % of comonomer 2-hydroxypropylmethacrylate and 1, 1.5, 2 and 3 mol % of ethyleneglycoldimethacylate were successfully synthesized. DSC thermograms of p(NIPA/HPMA) hydrogels showed two sharp peaks from melting of crystalline regions (first from temperature 153.90 °C to 156.49 °C and second from 156.98 °C to 160.73 °C). Also, they exhibited more than one peak with weak intensity from glass transition of amorphous regions (first in the range of 63.41–64.79 °C, second in the range of 77.53–86.47 °C and third at 134.81–131.76 °C). DSC results confirmed that p(NIPA/HPMA) hydrogels are convenient for sterilizing and enable biomedical application as drug carriers. All p(NIPA/HPMA) hydrogels show temperature sensitivity, and the exhibited swelling behavior is known as negatively thermo-sensitive. The fluid transport into the p(NIPA/HPMA) network at 18 °C and 38 °C exhibited an anomaly in the fluid transport mechanism, i.e., the swelling dynamics were controlled by both diffusion of fluid and macromolecular chain relaxation, and did not obey the Fickian law. The mathematical modeling of the swelling process indicates that hydrogels follow a second-order kinetics. The morphological and structural characteristics in the p(NIPA/HPMA) hydrogels with loaded acetaminophen were verified using SEM and FTIR methods. The loading efficiency of acetaminophen into the p(NIPA/HPMA) hydrogels is in the range of 92.16–96.06% and confirms excellent loading efficiency. The kinetic parameters of the acetaminophen release from the p(NIPA/HPMA) hydrogels at pH 7.4 and for hydrogel sample with 1 mol % of crosslinker at pH 2.2 correspond to Fickian diffusion, and p(NIPA/HPMA) hydrogels with 1.5, 2 and 3 mol % of crosslinker at pH 2.2 correspond to non-Fickian diffusion. Applied p(NIPA/HPMA) hydrogels could be suitable as acetaminophen carriers due to their superior thermosensitive properties. The in vitro acetaminophen delivery from the temperature-sensitive p(NIPA/HPMA) hydrogels proved that it might be interesting for further studies on extended acetaminophen delivery.

## 4. Materials and Methods

### 4.1. Reagents

*N*-Isopropyl acrylamide 99%, 2-hydroxypropylmethacrylate 96.5% and *α,α′*-azoisobutyronitrile 98% (Acros Organics, Morris Plains, NJ, USA); methanol 99.9% HPLC grade and ethyleneglycoldimethacylate 97% (Fluka Chemical Corp., Buchs, Switzerland); 4-Acetamidophenol ≥ 98% (Acros Organics N.V., Fair Lawn, NJ, USA); potassium bromide, KBr, for IR spectroscopy ≤ 100% (Merck KGaA, Darmstadt, DE, USA); methanol p.a. (Unichem, Belgrade, RS, USA); acetone (Centrohem, Belgrade, RS, USA).

### 4.2. Hydroges Synthesis

**Figure 12:**
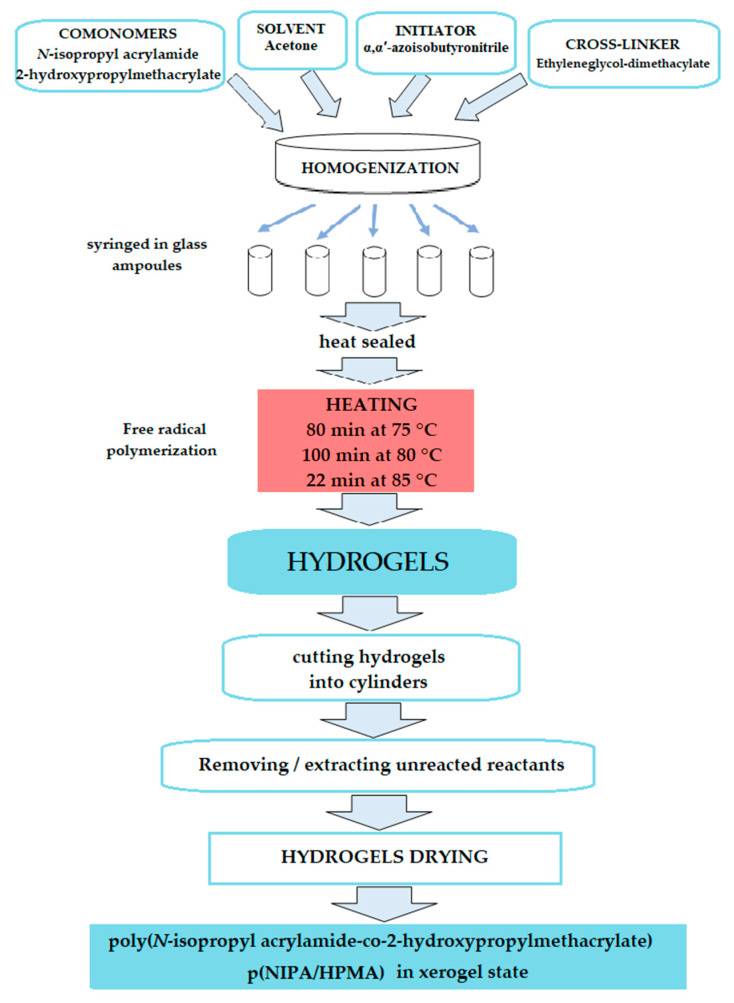
Flow chart of p(NIPA/HPMA) hydrogel polymerization process.

Hydrogel networks based of both comonomers *N*-isopropyl acrylamide and 2-hydroxypropylmethacrylate (10 mol % related to the quantity of NIPA monomer) were synthesized using the free radical polymerization method [9,43,44,47]. Ethyleneglycoldimethacylate (0.5, 1.0, 1.5, 2.0 and 3.0 mol % related to the total comonomers mass) was added to crosslinking reactants. Acetone was used to dilute reaction mixture, which was initiated by 2.7 mol % of α,α′-azoisobutyronitrile relative to the total comonomers mass. All samples after homogenization were syringed in glass ampoules, and afterward, they were heat sealed. The polymerization was performed as following: 80 min at 75 °C, 100 min at 80 °C and 22 min at 85 °C, until all unsaturated bonds of α,α′-azoisobutyronitrile were cleaved to produce radicals. Synthesized p(NIPA/HPMA) hydrogels were cut into discs (*d* × *l* = 5 × 2, *d*, mm, the diameter, and *l*, mm, the thickness) after separation from the glass ampoules. For the removal of unreacted reactants, hydrogels were extracted using methanol as solvent during 48 h. After that, hydrogels were subsequently immersed in methanol/distilled water solutions 100/0, 75/25, 50/50, 25/75, for a day, and 0/100% for 2 days, and then dehydrated to constant mass to reach the xerogel stage (at 40 °C). Figure 12 shows a flow chart of the polymerization process.

### 4.3. DSC Method

Thermal characteristics of the p(NIPA/HPMA) copolymers were examined using the differential scanning calorimetry (DSC) method. Hermetically sealed aluminum vessels with 3–5 mg of xerogels in powder state were heated in a nitrogen atmosphere from ambient temperature up to 180 °C (heating dynamics 10 °C/min^−1^). The sensitivity of the used instrument DSC Q20 (TA instruments, New Castle, DE, USA) was 10 mVcm^−1^. Standard calibration was accomplished by using indium.

### 4.4. Swelling Behavior

The swelling process of the p(NIPA/HPMA) hydrogels in the xerogel state was supervised gravimetrically at 18 °C (as the lower recommended room temperature) and 38 °C (as an inner body temperature) [46,47]. All samples were first measured, and after that, immersed in the fluid (pH = 7.4). At determined times, the hydrogels were taken out, their masses were measured, and then they were returned until constant mass was achieved. The percentage of swelling degree, α(%), was determined according to Equation (1) [45] and the equilibrium swelling degree, α_e_, was determined according to Equation (2) [9,21,60]:

(1) $$\alpha (\%)=\frac{m_{t}-m_{0}}{m_{0}}\times 100$$

(2) $$\alpha_{e}=\frac{m_{e}-m_{0}}{m_{0}}$$

*m_t_*—the mass of the hydrogel swollen at the time *t*, *m_e_*—the mass of the hydrogel swollen at equilibrium, *m*_0_—the initial mass of xerogel.

#### 4.4.1. Temperature Sensitivity

The temperature sensitivity of p(NIPA/HPMA) hydrogels was supervised in a water bath (Sutjeska, Belgrade, RS, USA) during heating from 5 to 60 °C. The measured p(NIPA/HPMA) xerogels were swollen in distilled water for 24 h at determined temperatures to reach equilibrium, the mass of the swollen hydrogel, *m_t_*, was measured, and after that, the hydrogels were immersed into distilled water. The percentage of swelling degree was evaluated by Equation (1).

#### 4.4.2. Kinetic Analysis

The nature of the fluid diffusion process into the analyzed p(NIPA/HPMA) hydrogel samples, throughout the swelling and acetaminophen release processes, was evaluated using Fick’s law (3) in order to calculate the fractional sorption, *F*, and fitting the experimental results [61,62]:

(3) $$F=\frac{M_{t}}{M_{e}}=k\times t^{n}$$

*M_t_*—content of the absorbed liquid at the time *t*, *M_e_*—content of the absorbed liquid in the equilibrium, *k*, min^1/n^—the characteristic kinetic constant, n—the diffusion exponent (indicative of the transport mechanism). If the value of n < 1/2, the mechanism of fluid transport obeys the Fickian diffusion; a value of 1/2 < n < 1 designates sigmoidal (anomalous), or non-Fickian diffusion characterized by the water-sorption process. If the value of n = 1/2, the diffusion grade is significantly lesser than the relaxation of the macromolecular chains (Case I), while n = 1 points to the diffusion of fluid being significantly faster in relation to the polymer chains relaxation (Type II, Case II). If the value of n > 1, the relaxation of polymer chains controls swelling, and this case is named Super Case II, Type III, or Case III.

By logarithmization of Equation (3), Equation (4) is obtained [61,62]:

(4) ln F=ln⁡MtMe=ln⁡k+n·ln⁡t

The values of the exponents *n* and *k* are calculated from the slope and intercept of the linear relationship between ln*F* and ln*t*. The diffusion coefficient *D* is calculated for the initial phase of swelling to 60% from Equation (5) [61,62]:

(5) $$\frac{M_{t}}{M_{e}}=4\cdot {\left(\frac{Dt}{\pi l^{2}}\right)}^{0.5}$$

*l*—the dried sample thickness (cm), and *D*—the diffusion coefficient (cm^2^/min). By taking the logarithm of Equation (4), Equation (6) is obtained (the linear relationship between ln(*M_t_*/*M_e_*) and ln*t*):

(6) $$\ln\frac{M_{t}}{M_{e}}=\left(\frac{{4D}^{0.5}}{\pi^{0.5}l}\right)+\frac{1}{2}\ln t$$

The diffusion coefficient is calculated from the intercept of the linear relationship between ln(*M_t_*/*M_e_*) and ln*t*, where *t* is the time for which the hydrogel absorbs half of the total amount of fluid [61,62].

#### 4.4.3. The Order of the Swelling Reaction

The order of the swelling process was calculated using the results of swelling kinetics, for synthesized p(NIPA/HPMA) hydrogels [60,63,64]. The normalized swelling ratio at time *t* is calculated applying the following expression (7):

(7) $$\ln\frac{\alpha_{e}-\alpha_{t}}{\alpha_{e}}=-Kt$$

If the swelling process obeys first-order kinetics, this dependence (7) is linear with a slope of −K. Should this not happen, it is than checked whether the swelling process follows a second-order reaction or not. The swelling kinetics obey a second-order reaction described by Robinson–Schott’s Equation (8) [63,64,65]:

(8) $$\frac{t}{\alpha_{t}}=\frac{1}{K\alpha_{e}{}_{}^{2}}+\frac{t}{\alpha_{e}}$$

The constructed plot of *t/α_e_* versus *t*, according to the expression (8), should be linear. The normalized equilibrium swelling ratio, the second-order rate constant as an intercept of 1/*Kα_e_* and the linear correlation coefficient are calculated using the experimental results by expressions 7 and 8.

### 4.5. Acetaminophen Loading into the p(NIPA/HPMA)

Acetaminophen solution, 40 mg/cm^3^, was prepared by dissolving the drug in a solvent mixture of methanol/distilled water, 80/20 *v*/*v*, at a room temperature. With the aim of loading the drug into the p(NIPA/HPMA) hydrogels, measured samples (at about 0.10 g) in the xerogel state were swollen in the prepared acetaminophen solution for 48 h at 5 °C (stored in refrigerator) to reach equilibrium state. The acetaminophen maximum available amount for loading into the p(NIPA/HPMA) hydrogel was 500 mg/g_xerogel_. Swollen p(NIPA/HPMA) hydrogels with loaded acetaminophen were washed using distilled water to remove excess acetaminophen, then dried to remove water and methanol, and measured in the xerogel state. Acetaminophen loading efficiency, *η*(%), was calculated using Equation (9) [43,44,45]:

(9) $$\eta (\%)=\frac{L_{g}}{L_{u}}\times 100$$

*L_g_* (mg/g_xerogel_)—the acetaminophen loaded mass into p(NIPA/HPMA) hydrogel sample,

*L_u_* (mg/g_xerogel_)—the acetaminophen maximum available mass for loading into p(NIPA/HPMA) hydrogel sample.

### 4.6. In Vitro Acetaminophen Release Study

Acetaminophen release from the p(NIPA/HPMA) carrier with loaded drug was studied in vitro under simulated physiological conditions. The p(NIPA/HPMA) hydrogel samples with loaded acetaminophen were overflowed with 7 cm^3^ of alkaline or acidic fluids (either sodium hydroxide at pH 7.4 or hydrochloric acid at pH 2.2) and thermostated at 38 °C. Acetaminophen release was monitored during 24 h. For RP HPLC analysis, aliquot parts of the flud with released acetaminophen (100 µL) were sampled in fixed times, and methanol was added and then filtered for RP HPLC analysis through a cellulose membrane filter (0.45 μm). The HPLC Agilent 1100 Series with diode-array detector, DAD 1200 Series (Agilent Technologies, Santa Clara, CA, USA) and column Supelcosil LC-18-DB, Hypersil GOLD 25 cm × 4.6 mm, 5 μm particle size (Supelco, Sigma-Aldrich Chemie GmbH, Taufkirchen, Germany) devices were used at a working column temperature of 25 °C, with methanol for HPLC as eluent (flow 1 cm^3^/min), 20 μL injected volumes of each sample, at detector wavelength 220 nm for the acetaminophen.

Aimed at the calibration curve plot construction, first a set of standard solutions containing known concentrations of acetaminophen analyte were made ready for use. Then, they were filtered over a cellulose membrane filter (0.45 μm) for RP HPLC analysis. Agilent ChemStation software was used for analysis of each recorded chromatograms. The linear part of the calibration curve ranged from 0.005 to 0.800 mg/cm^3^ with the linear correlation coefficient *R*^2^ = 0.9994, and the quantity of acetaminophen released from p(NIPA/HPMA) hydrogels was calculated applying Equation (10):

(10) $$c_{acetaminophen}=\frac{A-165.6}{14,756.4}$$

where *c*_acetaminophen_, mg/cm^3^, is the acetaminophen amount and *A*, mAUs, is the peak area.

### 4.7. Characterization

#### 4.7.1. FTIR Method

Acetaminophen, both samples in the xerogel state of the obtained pristine p(NIPA/HPMA) copolymer and p(NIPA/HPMA) with entrapped acetaminophen, were turned into powder form with an Amalgamator (WIG-L-Bug, Dentsply RINN, a Division of Dentsply International Inc., Smile Way, York, PA, USA). All samples in the powder state (0.9 mg) with the 150 mg of KBr were vacuumed and pressed under 200 MPa to create transparent tablets. FTIR spectra were scanned (16 scans) using an FTIR spectrophotometer, Bomem Hartmann & Braun MB-series (Hartmann & Braun, Baptiste, CA, USA), at the resolution of 2 cm^−1^ in the near IR range (4000–400 cm^−1^ wavenumbers). Win-Bomem Easy software was used for FTIR spectra analysis.

#### 4.7.2. Freeze-Drying of Hydrogels

Freeze-drying of synthesized p(NIPA/HPMA) hydrogels without and with loaded acetaminophen was carried out on a LH Leybold, Lyovac GT2 device (Frenkendorf, Switzerland). The p(NIPA/HPMA) hydrogels swollen to equilibria were firstly rapidly frozen at −22 °C over 24 h. Then, absorbed fluid was removed at −30 °C at a high vacuum (0.05 kPa) over 14 h in the sublimation subphase. In the next subphase, isothermal desorption, the hydrogels were kept at 20 °C through 6 h at the same vacuum (0.05 kPa). Freeze-dried p(NIPA/HPMA) hydrogels without and with loaded acetaminophen were packed and kept in the refrigerator.

#### 4.7.3. Scanning Electron Microscopy

The morphology of selected p(NIPA/HPMA) hydrogels freeze-dried in the equilibrium swollen state, pure and with acetaminophen loaded, was observed by scanning electron microscopy. Firstly, all samples were covered with a gold/palladium alloy (85/15) under vacuum conditions with a sprayer, JEOL Fine Coat JFC 1100E Ion Sputter (JEOL Co., Tokyo, Japan), and, after that, scanned on a JEOL Scanning Electron Microscope JSM-5300 (JEOL Ltd., Tokyo, Japan).

### 4.8. Statistical Analysis

The data obtained in the experiments of swelling and in vitro acetaminophen release were evaluated using one-way analysis of variance (ANOVA) using the SPSS statistical package. Statistical differences results (*p* ≤ 0.05) were considered significant. All results were determined to be within the 95% confidence level for reproducibility.

## 5. Patents

Granted patent RS53220B: Ilić-Stojanović, S.; Nikolić, Lj.; Nikolić, V.; Petrović, S.D.; Stanković, M. Process for synthesis of thermosensitive hydrogels and pharmaceutical applications, The Intellectual Property Office of the Republic of Serbia, https://worldwide.espacenet.com/patent/search/family/046025219/publication/RS53220B?q=RS53220B (accessed on 1 April 2023).

## Figures and Tables

**Table 1:** The melting temperatures (*T*_m_), the melting enthalpy (ΔH_m_) and the glass transition temperatures (*T*_g_), for the obtained p(NIPA/HPMA) xerogels with 1.0, 2.0, and 3.0 mol % of EGDM.

Poly(*N*-isopropyl acrylamide-*co*-2-hydroxypropylmethacrylate)	Melting Transition Temperature, °C		Melting Enthalpy, J∙g^−1^	Glass Transition Temperature, T_g_, °C		
p(NIPA/HPMA) with	*T*_*m*1_	*T*_*m*2_	ΔH_m_	*T*_*g*1_	*T*_*g*2_	*T*_*g*3_
1 mol % of EGDM	153.90	159.07	5.99	63.41	77.53	131.76
2 mol % of EGDM	156.49	160.73	9.85	64.79	85.17	134.16
3 mol % of EGDM	154.16	156.98	2.79	64.23	86.47	-

**Table 2:** The equilibrium swelling degree (α_e_) of p(NIPA/HPMA) hydrogels at 18 °C and pH 7.4 and kinetic parameters of the fluid diffusion (*n, k* and *D*).

p(NIPA/HPMA) * with	Equilibrium Swelling Degree, α_e_	Diffusion Exponent, *n*	Kinetic Constant, *k* × 10^2^, min^−1/2^	Linear Correlation Coefficient, R^2^	Diffusion Coefficient, *D*, cm^2^·min^−1^
18 °C					
1 mol % of EGDM	25.960	0.822	1.998	0.981	8.998 × 10^−6^
1.5 mol % of EGDM	14.748	0.761	1.105	0.991	2.703 × 10^−6^
2 mol % of EGDM	10.143	0.681	0.989	0.993	0.379 × 10^−6^
3 mol % of EGDM	6.943	0.584	0.971	0.996	0.691 × 10^−6^
38 °C					
1 mol % of EGDM	6.662	0.984	3.323	0.996	0.784 × 10^−5^
1.5 mol % of EGDM	4.196	0.701	2.601	0.964	0.676 × 10^−5^
2 mol % of EGDM	3.429	0.692	2.251	0.981	1.399 × 10^−5^
3 mol % of EGDM	2.986	0.611	2.292	0.936	3.102 × 10^−5^

* p(NIPA/HPMA)—poly(*N*-isopropyl acrylamide-*co*-2-hydroxypropylmethacrylate) hydrogel.

**Table 3:** The experimentally and calculated normalized equilibrium swelling degree and kinetic parameters of the first- and second-order reaction at 18 °C and 38 °C.

p(NIPA/HPMA) * with	Equilibrium Swelling Ratio, α_e_ (exp)	Equilibrium Swelling Ratio, α_e_ (I-order)	Rate Constant (I-order), *K*·10^3^, min^−1^	Linear Correlation Coefficient (I-order), R^2^	Equilibrium Swelling Ratio, α_e_ (II-order)	Rate Constant (II-order), *K*·10^3^, min^−1^	Linear Correlation Coefficient (II-order), R^2^
18 °C							
1 mol % of EGDM	25.960	27.214	2.35	0.982	26.113	26.286	0.999
1.5 mol % of EGDM	14.748	15.972	3.96	0.971	14.986	5.691	0.999
2 mol % of EGDM	10.143	11.266	3.23	0.934	10.534	13.118	0.999
3 mol % of EGDM	6.943	8.32	3.98	0.961	7.112	4.882	0.999
38 °C							
1 mol % of EGDM	6.662	8.012	9.36	0.989	6.892	6.141	0.999
1.5 mol % of EGDM	4.196	4.949	7.33	0.991	4.464	6.841	0.999
2 mol % of EGDM	3.429	3.833	7.18	0.996	3.678	5.654	0.999
3 mol % of EGDM	2.986	3.524	8.59	0.991	3.274	4.991	0.998

* p(NIPA/HPMA)—poly(*N*-isopropyl acrylamide-*co*-2-hydroxypropylmethacrylate) hydrogel.

**Table 4:** Characteristic absorption band maximum positions for p(NIPA/HPMA), acetaminophen, p(NIPA/HPMA) with loaded acetaminophen and value of wavenumbers shifts after drug incorporation.

Wavenumber of Functional Group, cm^−1^			Functional Group	Shifts in Relation to the FTIR Spectra, cm^−1^	
p(NIPA/HPMA) *	Acetaminophen	p(NIPA/HPMA) with Acetaminophen		p(NIPA/HPMA)	Acetaminophen
3438		3436	ν(OH)	−2	
3319	3324	3326	ν(NH)	+7	+2
	3162	-	ν(Ar-OH)		-
	3035	-	ν(C-H) Ar		-
2973	2929	2974	ν_as_(CH_3_)	+1	
2933		2934	ν_as_(CH_2_)	+1	
2876	2880	2877	ν_s_(CH_3_)	+1	−3
1728		1720	ν(C=O) ester	−8	
1650	1654	1656	ν(C=O) amide I	+6	+2
	1610	1610	ν(C=C) Ar		0
1544	1564	1560	δ(N-H) amide II	+16	−4
	1507	1507	ν(C=C) Ar		0
1460, 1387	1442	1453, 1369	δ(OH)	−7, −9	+11
1367		1369	δ(CH)-isopropyl	+2	
1306		-	ν_s_(C-N) amide III		
	1370, 1328	1327	δ_s_(CH_3_)		−1
	1260	1260	ν(C-O)		
	1227	1243	ν(NCH)		+16
1173	1172	1172	ν(CN)	−1	0
1131		1131	ν_s_(C-O)	0	
837	837	837	γ(CH)	0	0
	808	797	γ(CH)		−11
674	686	688	γ(OH)	+14	+2

* p(NIPA/HPMA)—poly(*N*-isopropyl acrylamide-*co*-2-hydroxypropylmethacrylate) hydrogel.

**Table 5:** The amount of loaded acetaminophen (*L*_g_) into the series of p(NIPA/HPMA) hydrogels and acetaminophen loading efficiency (*η*_acetaminophen_).

p(NIPA/HPMA) * with	Amount of Loaded Acetaminophen, *L*_g_, mg/g_xerogel_	Acetaminophen Loading Efficiency, *η*_acetaminophen_, %
1 mol % of EGDM	480.34	96.06
1.5 mol % of EGDM	460.84	92.16
2 mol % of EGDM	467.63	93.52
3 mol % of EGDM	480.81	96.14

* p(NIPA/HPMA)—poly(*N*-isopropyl acrylamide-*co*-2-hydroxypropylmethacrylate) hydrogel.

**Table 6:** Quantity of released acetaminophen from p(NIPA/HPMA) hydrogels (in mg/g and %) and kinetic parameters of diffusion (*n*, *k* and *D*) at 38 °C, pH 2.2 and pH 7.4.

Poly(*N*-isopropyl acrylamide-*co*-2-hydroxypropylmethacrylate) with	Quantity of Released Acetaminophen, mg/g_xerogel_%		Diffusion Exponent, *n*	Kinetic Constant, *K,* min^−1/2^	Linear Correlation Coefficient, *R*^2^	Diffusion Coefficient, *D*, cm^2^/min
pH 7.40						
1 mol % of EGDM	492.77	98.56	0.297	0.641	0.972	3.79 × 10^−3^
1.5 mol % of EGDM	484.11	96.82	0.325	0.694	0.997	3.74 × 10^−3^
2 mol % of EGDM	471.59	94.32	0.297	0.756	0.972	4.48 × 10^−3^
3 mol % of EGDM	455.58	91.12	0.375	0.639	0.944	3.21 × 10^−3^
pH 2.2						
1 mol % of EGDM	480.16	96.03	0.486	0.747	0.881	4.38 × 10^−3^
1.5 mol % of EGDM	474.78	94.96	0.541	0.737	0.846	4.26 × 10^−3^
2 mol % of EGDM	465.66	93.12	0.533	0.722	0.961	4.10 × 10^−3^
3 mol % of EGDM	451.43	90.32	0.662	0.666	0.961	3.48 × 10^−3^

**Table 7:** The comparison of the acetaminophen release from different polymeric carriers.

Polymeric Carrier	Experimental Conditions and Released Acetaminophen	Mechanism of Acetaminophen Diffusion	Reference
Poly(*N*-isopropyl acrylamide-*co*-2-hydroxypropylmethacrylate) with ethyleneglycoldimethacrylate	98.56% at pH = 7.4, and 96.03% at pH = 2.2 during 24 h at 38 °C.	At pH = 7.4—Fickian diffusion. At pH = 2.2—non-Fickian diffusion (gels with 1.5, 2 and 3 mol % of EGDM).	[current study]
Poly(*N*-vinylpyrrolidinone-*co*-acrylic acid) (with 30 wt % AA) and polyethylene glycol 600 dimethacrylate	At pH 2 approx. 24 h pH 6.8 approx. 5 h pH 9 approx. 5 h at 37 °C.	-	[36]
Sodium alginate and *N*-isopropyl acrylamide crosslinked with *N,N*’methylenebisacrylamide	At 37 °C in pH 2.2 for 9 days ~90%.	The first-order kinetic. Non-Fickian (anomalous) diffusion.	[37]
Poly(acrylamide-*co*-itaconic acid) crosslinked with *N,N*-methylenebisacrylamide	At 37 °C during 8 h: at pH = 2.2 ~20–55% pH = 4.5 ~90–99% pH = 6.8 ~90–99%.	Slow drug release under acidic conditions and rapid release at higher pH value. Fickian diffusion.	[38]
Polyacrylamide with 4-[(4-methacryloyloxy) phenylazo] benzenesulfonic acid and *N,N*′-hexylenebismethacrylamide	The photoregulated release at 353 nm for 120 min of irradiation, a total of 83.6% was released in aqueous HEPES buffer pH 7.16.	-	[39]
Poly(2-hydroxyethyl methacrylate-*co-N*-vinyl-2-pyrrolidone) with *N,N’*-methylenebisacrylamide	at 37 °C and SGF pH = 1.2, SIF pH = 7.2 ~95% during 240 min (4 h).	The tablet from the polymer NVP3 crumbled within an hour of immersion, resulting in burst release. Non-Fickian (anomalous) diffusion.	[40]
Hydroxypropyl cellulose with polyacrylamide, 25/75 wt %	In deionized water, pH 7 in phosphate buffer, pH 7.38 at 35 °C, 37 °C, 39 °C, during 6 h.	Drug was crystallized on the gel surface. Fickian diffusion.	[41]
Polyurethane nanocomposite hydrogels PU/PEG 4000 with 1% of organofillized montmorillonite (Cloisite^®^ 30B)	In water for 24 h at 23 °C and 37 °C.	Easier release from nanocomposites than from a pure hydrogel matrix. Non-Fickian (anomalous) diffusion.	[42]
Poly(N-isopropylacrylamide-acrylamide) with *N,N*-methylenbisacrylamide, and *N,N,N,N*-tetra-methylethylenediamine	pH= 7.4 at 27 °C, 32 °C, 41 °C, 44 °C ± 0.1 °C	The pore mechanism of drug transport.	[43]
Poly(vinyl alcohol) with nanofibrillated cellulose, (NFC)/PVA, and 2,2,6,6-tetramethylpiperidine-*N*-oxyl-oxidized nanofibrillated cellulose (TNFC)/PVA without any chemical linkers	At 37 °C and phosphate buffer, 14% from (NFC)/PVA and about 28% from (TNFC)/PVA over 144 h (6 days).	Diffusion-controlled and burst release, with small fractions of relaxation-induced and prolonged-diffusional release.	[44]

## Data Availability

Not applicable.
